# Attenuation Value in Adrenal Incidentalomas: A Longitudinal Study

**DOI:** 10.3389/fendo.2021.794197

**Published:** 2021-12-02

**Authors:** Filippo Ceccato, Irene Tizianel, Giacomo Voltan, Gianmarco Maggetto, Isabella Merante Boschin, Emilio Quaia, Filippo Crimì, Carla Scaroni

**Affiliations:** ^1^ Department of Medicine (DIMED), University of Padova, Padova, Italy; ^2^ Endocrine Disease Unit, University-Hospital of Padova, Padova, Italy; ^3^ Department of Neuroscience (DNS), University of Padova, Padova, Italy; ^4^ Department of Surgical Oncological and Gastroenterological Sciences (DiSCOG), University of Padova, Padova, Italy; ^5^ Institute of Radiology, University-Hospital of Padova, Padova, Italy

**Keywords:** adrenal incidentaloma, autonomous cortisol secretion, attenuation value, computed tomography, Hounsfield Unit

## Abstract

**Context:**

A tendency to grow has been reported in adrenal incidentalomas. However, long-term data regarding attenuation value, a measure of lipid content, are not available.

**Aim:**

This study aims to collect radiological data (diameter in mm and attenuation value in Hounsfield units, HU) with computed tomography (CT) in adrenal incidentalomas, in order to compare baseline characteristics with the last follow-up imaging.

**Design:**

This is a longitudinal study which included patients with a new diagnosis of adrenal incidentaloma, evaluated from January 2002 to June 2020.

**Setting:**

Referral University-Hospital center.

**Patients:**

Two hundred seventy-seven patients with 355 different cortical adenomas (baseline group) were evaluated at the first outpatient visit; the follow-up cohort consists of 181 patients with 234 adenomas (12–175 months after baseline). Inclusion criteria were conservative management and radiological features able to minimize malignancy or risk of progression.

**Main Outcome Measure:**

CT modification according to endocrine function: autonomous cortisol secretion (ACS) if cortisol >50 nmol/L after 1-mg dexamethasone test (DST).

**Results:**

At baseline CT, mean diameter was 18.7 mm and attenuation value was 0.8 HU (higher in ACS, 66 cases >10 HU), without modification in early imaging (12–36 months). The size increased over time (*r* = 0.289), achieving the largest differences after at least 60 months of follow-up (mean diameter, +2 mm; attenuation value, −4 HU), combined with a reduction in the attenuation value (*r* = −0.195, especially in patients with ACS). Lipid-poor adenomas (>10 HU) presented a reduced cortisol suppression after 1-mg DST, an increase in size and the largest decrease in attenuation value during follow-up. Univariate analysis confirmed that larger adenomas presented reduced suppression after DST and increase in size during follow-up.

**Conclusions:**

Growth is clinically modest in adrenal incidentaloma: the first follow-up CT 5 years after baseline is a reasonable choice, especially in ACS. Mean density is increased in patients with ACS and overt hypercortisolism. Mean density reduces during follow-up in all adrenal adenomas, suggesting an increase in lipid content, especially in those with ACS.

## Introduction

Adrenal incidentalomas are incidentally discovered masses, during a radiological study not performed for the suspicion of an adrenal-related disorder, as arterial hypertension, hypokalemia, early-onset diabetes, metabolic syndrome, or osteoporotic fractures (1). They represent an important challenge for differential diagnosis among several adrenal and extra-adrenal diseases (2, 3). The detection of adrenal incidentalomas is increasing over the last years, up to 10%–15% in subjects over 70 years old (2, 4), due to the large availability of imaging medical equipment in routine clinical practice, such as computed tomography (CT) and magnetic resonance (MR) (5). In most cases, adrenal incidentalomas are benign nonfunctioning cortical adenomas that do not require further studies or surgical/medical treatment (1).

Considering the endocrine function, most guidelines suggest to rule out at the baseline visit pheochromocytoma, primary aldosteronism, and Cushing’s syndrome (CS) (1, 2). A consistent cohort of patients present a subclinical autonomous cortisol secretion (ACS), without the full-blown clinical picture of overt hypercortisolism (6, 7). Several studies reported a progression from a nonfunctioning adrenal incidentaloma (NFAI) to ACS in up to 11% of cases (8–10), especially in larger adenomas (11, 12).

The guidelines of the European Society of Endocrinology (ESE) in collaboration with the European Network for the Study of Adrenal Tumours (ENS@T) define benign adrenal incidentalomas as those with low attenuation value, homogeneous texture, and diameter <4 cm (1) [the diameter has been confirmed in a retrospective study with 233 patients (13)]. In clinical practice, a growth tendency has been observed in adrenal incidentalomas: an increase of >0.5 cm has been described in 20 out of 77 patients in a 5-year study (14). Larger cohorts with shorter follow-up (median 2 years) reported an increase >1 cm in 12/229 (9) and 25/139 patients (15). A positive correlation between adenoma diameters and serum cortisol levels after dexamethasone suppression test (DST) has been observed (16). A recent systematic review reported a minimal growth (2 mm in 4 years), an increase of >1 cm was observed in 2.5% of cases (17).

The calculation of tissue attenuation or density values, measured in Hounsfield units (HU), is the assessment of X-ray absorption during CT (18, 19). An inverse relationship between lipid content and the attenuation value obtained with unenhanced CT is described in adrenal adenomas (1, 2, 20): a density of <10 HU indicates a lipid-rich adenoma with 71% sensitivity and 98% specificity (18). At the best of our knowledge, scarce data have been reported regarding the modification in attenuation value during follow-up. Only one study by Hammarstedt et al. reported in 2012 a slight increase in the lipid content of adrenal incidentalomas after 2 years of follow-up; nonetheless, cortisol secretion was not considered.

In the present study, we investigated the radiological changes (diameter and attenuation value) in a cohort of patients with adrenal incidentalomas, according to their cortisol secretion, after a long-term follow-up.

## Materials and Methods

### Patients

This longitudinal study included patients with a new diagnosis of adrenal incidentaloma, evaluated from January 2002 to June 2020. A dedicated query (incidental findings of an adrenal mass) was used in the web-based Padova University-Hospital database to extract the initial cohort of patients with a regular follow-up in the Endocrinology Unit (*n* = 460 patients). Then specific inclusion criteria for this study were:

- Rule out of malignancy and overt hormonal secretion. Patients with any active malignancies (not only adrenal) or clear signs/symptoms of endocrine excess were excluded. Normal serum aldosterone/renin ratio [after appropriate interfering drug wash-out (13)] and urinary fractionated metanephrine level (14) were used to rule out primary aldosteronism and pheochromocytoma. Overt adrenal CS was searched in all patients with clinical signs/symptoms consistent with endogenous hypercortisolism. In case of unsuppressed serum cortisol after 1-mg DST (>50 nmol/L, performed in all patients), urinary free cortisol (UFC) and late-night salivary cortisol (LNSC) were evaluated with a home-brew LC-MS/MS method previously described (21, 22).
- Radiological evidence at CT of a benign adrenal incidentaloma. All images of an unenhanced abdominal CT were available in the database of the University-Hospital of Padova. If attenuation value was not sufficient to indicate a lipid-rich adenoma in unenhanced CT (HU <10), a contrast-enhanced CT or a MR was performed. Adrenal adenoma was confirmed in case of absolute washout of >60% or a relative washout of >40% in the delayed images (15 min for the venous phase) during contrast-enhanced CT or a signal drop in the out-of-phase images of >20% during MR scan (23). Same criteria were used in the follow-up study.
- CT image consistent with a discrete cortical adrenal nodule. All cases of adrenal hyperplasia (one or both adrenals massively enlarged by the presence of multiple macronodules >1 cm) and adrenal myelolipoma (diagnosed by its peculiar imaging) were excluded (20).
- Conservative management of the adrenal incidentaloma during the follow-up. All surgical cases were excluded from baseline evaluation, in order to reduce selection bias. Surgical management was proposed in patients with suspected malignant features (24), adrenal secretion, or incidentalomas with a rapid increase in size (>5 mm in 6 months was considered clinically significant) after a multidisciplinary evaluation (3).

According to the aforementioned criteria, we selected 277 patients (157 females, 57%), median age 66 years old (range 29,–86). Two hundred eight one monolateral adrenal incidentaloma, 62 patients two adenomas, five patients three different adenomas, and two patients four adenomas (bilateral incidentaloma in 64 patients). Three hundred fifty-five different discrete cortical adenomas were considered for the baseline evaluation. A follow-up CT was available after at least 12 months (median interval 52 months, range 12–175) in 181 patients with 234 adenomas.

We evaluated also CT scan 8 in patients with a clinical and radiological diagnosis of adrenal CS (collection period 2014–2018). Evidence of monolateral adenoma, full-blown clinical picture at diagnosis, increased UFC and LNSC levels, unsuppressed serum cortisol after 1-mg DST, baseline ACTH <10 pg/m, histological confirmation of adenoma, and cortisol insufficiency after surgery were the criteria adopted to confirm adrenal hypercortisolism.

Endocrine data collected at baseline and during the last follow-up visits were serum cortisol after 1-mg DST, morning ACTH, and UFC levels. ACS was defined in case of cortisol secretion of >50 nmol/L after 1-mg DST; otherwise, incidentalomas were considered NFAI. Dexamethasone levels were measured with a home-made liquid-chromatography tandem-mass spectrometry method in all DST test (from 2014), previously described: dexamethasone levels <4.5 nmol/L were considered insufficient and 1-mg test was discarded (25). Clinical data collected included gender, age, body weight and height (to calculate BMI), blood pressure, glycated hemoglobin (HbA1c), presence of cortisol-related comorbidities as hypertension (systolic or diastolic blood pressure >130/90 mmHg or antihypertensive treatment), diabetes mellitus (increased fasting blood glucose or HbA1c, antidiabetic treatment), dyslipidemia, osteoporosis (lumbar or femoral t score <−2.5 or clinical evidence of frailty fractures).

Between 2002 and 2009, the CT scans were performed with a 16-slice scanner (Somatom Emotion™; Siemens Healthineers, Erlangen, Germany), craniocaudal image acquisition with 120 kV tube voltage, 225 mAs effective dose, 0.5 s rotation time, 0.75 mm detector collimation, and 0.8 pitch. Slice thickness was 1.5 mm for unenhanced acquisitions; all images analyzed had a soft-tissue reconstruction with a 30B kernel.

After 2009, all CT examinations were performed with a 128-slice scanner (Somatom Definition™; Siemens Healthineers, Erlangen, Germany), craniocaudal image acquisition with 120 kV tube voltage, 250 mAs effective dose, 0.5 s rotation time, 0.6 mm detector collimation, and 0.75 pitch. The slice thickness for unenhanced scans was 1.5 mm, and the reconstruction kernel was 30B. All CT scanners were calibrated every morning before the first patient, according to the University-Hospital of Padova standard procedures and were maintained according to the manufacturer’s specifications.

CT images were reviewed on a dedicated workstation by one endocrinologist and one radiologist expert in abdominal imaging (GM and FCr), blinded to clinical and biochemical data of the patients. Discrepancies (maximal diameters, difference in results higher than standard error of mean, selected area) were resolved by discussion and, if in case of disagreement, a third senior radiologist (EQ) was involved. The diameter of the adenoma was considered the mean of the two perpendicular maximal diameters on an axial plane. Mean attenuation values of the adenoma (HU^m^) was obtained by the average of three measurements with a circular or ovoid region of interest (ROI) cursors performed in the axial slice at unenhanced CT with the largest diameters of the lesion. Each ROI placed over the lesion included at least two-thirds of the area except edges to minimize partial volume effects; necrotic, cystic, hemorrhagic, and calcified areas within the ROI were avoided if possible. The same acquisition protocol was used to collect data in all CT scan (baseline and last follow-up available).

Our study complies with the Strengthening the Reporting of Observational Studies in Epidemiology (STROBE) statement and guideline (26).

The Ethics Committee of Padova University Hospital (Comitato Etico per la Sperimentazione Scientifica) approved the study (protocol No. 53401-2021). The clinical data were obtained from the web-based database of Padova University Hospital, in the form of electronic case reports or records.

### Statistical Analyses

Proportions and rates were calculated for categorical data. Continuous data were reported as means and standard deviation (SD), median and interquartile range (IQR), or difference (follow-up *versus* baseline, termed Δ). Groups were compared with the chi-square test for categorical variables (the raw *p*-values were adjusted with the Bonferroni method to take multiple comparisons into account), and with paired Student’s *t*-test for quantitative variables. From a clinical perspective, data from the last visit available was compared with the baseline consultation, and then the follow-up was divided in short (12–36 months), intermediate (37–60 months), and long term (>60 months).

Analysis of variance (ANOVA) was computed to observe variance data; *post-hoc* Dunnett’s test for multiple comparison procedure was used after a significant ANOVA, using the first quartile of mean diameter as a control group.

The SPSS 24 software package for Windows (SPSS, Inc., Chicago, IL, USA) was used to manage the database and perform the statistical analysis. The significance level was set at *p* < 0.05 for all tests. All data analyzed during this study are included in the data repositories of the University of Padova - Research Data UniPD (27).

## Results

**Figure 1:**
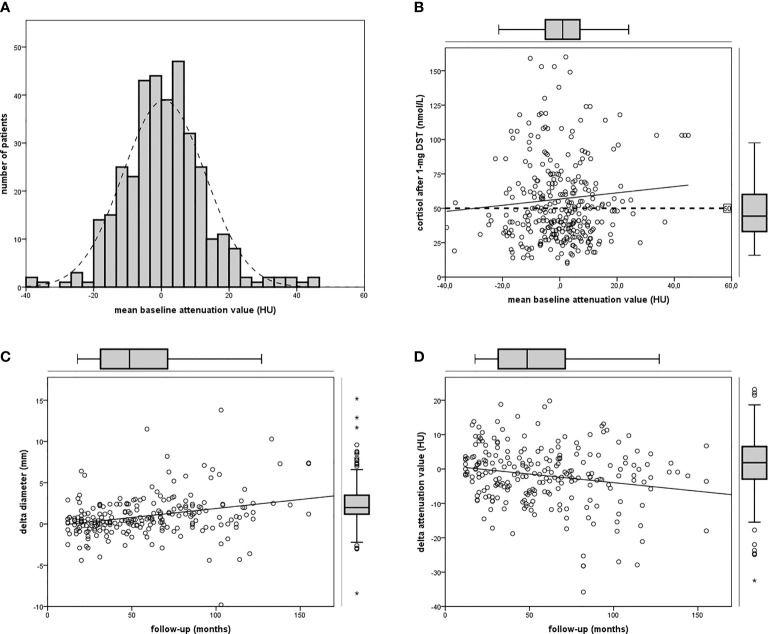
Attenuation value in the cohort of patients. **(A)** Frequency histogram of mean attenuation value; **(B)** linear regression between attenuation value and cortisol after 1-mg DST (*n* = 355); **(C)** linear regression between follow-up and Δ diameter (*n* = 234); **(D)** linear regression between follow-up and Δ attenuation (pn = 234).

According to our definition, 44% of patients presented ACS (at baseline 122 out of 277, at last visit 80 out of 181). Patients with ACS were older than those with NFAI, and most of the women were postmenopausal (only six still had periods). Hypertension was diagnosed in 77% (214 out of 277), dyslipidemia in 44% (123 out of 277), diabetes mellitus in 21% (58 out of 277), and osteoporosis in 11% (30 out of 277) at the baseline visit in all patients with adrenal incidentaloma. Clinical and radiological features in patients with NFAI or ACS are reported in **Table 1**. Attenuation value presented a symmetrical distribution and a positive correlation with cortisol after 1-mg DST (*r* = 0.219, *p* < 0.0001, depicted in **Figure 1**).

**Table 1:** Clinical and radiological data, presented as mean and standard deviation, or percentage if appropriate.

	NFAI	ACS	*p*-value
Age at diagnosis (years)	63.6 ± 4.9	66.6 ± 5.3	0.05
Gender female/male (% female)	81/74 (52%)	74/48 (61%)	0.081
BMI (kg/m^2^)	30.57 ± 4.81	28.48 ± 5.11	0.006
Basal ACTH (ng/L)	19.04 ± 11.18	13.69 ± 10.08	<0.0001
UFC (ULN)	0.53 ± 0.37	0.68 ± 0.21	0.007
HbA1c (mmol/mol)	41.69 ± 8.68	42.77 ± 9.21	0.33
Mean diameter (mm)	16.55 ± 5.81	20.94 ± 9.08	<0.0001
Attenuation value (HU^m^)	−0.84 ± 11.34	2.16 ± 12.97	0.027
HU^m^ follow-up > baseline (%)	48 (48%)	25 (31%)	0.013
HU^m^ follow-up < baseline (%)	53 (52%)	55 (69%)	
Hypertension (%)	118 (76%)	101 (83%)	0.216
Diabetes mellitus (%)	23 (15%)	28 (23%)	0.067
Dyslipidemia (%)	71 (46%)	52 (43%)	0.656
Osteoporosis (%)	11 (7%)	23 (19%)	0.001

NFAI, nonfunctioning adrenal incidentaloma; ACS, autonomous cortisol secretion; HU^m^, mean of Hounsfield Unit in unenhanced CT; UFC, urinary free cortisol; ULN, upper limit of normality; BMI, body mass index; HbA1c, glycated hemoglobin.

At baseline CT, mean diameter was 18.7 mm (SD ± 6.9 mm; range 8–42.1 mm; IQR 13.1–23.1 mm), and attenuation value was 0.8 HU^m^ (SD ± 12.2 HU^m^, range −39.5 to 44.9; IQR −6.4 to 7.5) in the whole cohort of 355 incidentalomas. Mean diameter was >40 mm in two patients (respectively 41 and 42.1 mm), attenuation value was >10 HU in 66 cases.

Eleven patients with NFAI developed ACS in the follow-up (8%); 19 patients with ACS at baseline reported serum cortisol <50 nmol/L after 1-mg DST at the last available visit. We observed during follow-up an increase in diameter and a reduction in attenuation values in the group of 234 patients with a control CT available, as reported in **Table 2**. Mean diameter at follow-up was 19.4 mm (SD ± 8.5 mm, range 8–42.2 mm, Δ +1 mm *versus* baseline); an increase ≥10 mm was observed in six incidentalomas. Age or age-based groups were not able to differentiate the radiological data reported, at baseline and during follow-up.

**Table 2:** Radiological data of patients with a follow-up CT scan.

Group	*n*, age at presentation (years)	Diameter baseline (mm)	Diameter follow-up (mm)	Δ Diameter (mm, 95% CI)	*p*-value *vs*. baseline	Attenuation value baseline (HU^m^)	Attenuation value follow-up (HU^m^)	Δ Attenuation value (HU^m^, 95% CI)	*p*-value *vs*. baseline
12–36 months of follow-up	78, 67.9 ± 8.4	18.13	18.29	+0.16 (−0.56 to 0.24)	0.42	−0.32	−0.08	−2.24 (−1.87 to 1.38)	0.756
37–60 months of follow-up	55, 66.6 ± 9.8	18.14	18.98	+0.84 (0.3–1.38)	0.003	0.53	−1.31	−1.83 (−1.87 to 0.13)	0.066
>60 months of follow-up	101, 66.6 ± 8.4	18.74	20.38	+1.65 (1.04–2.25)	<0.0001	2.82	−0.73	-3.55 (−5.5 - -1.59)	<0.0001
Baseline CT *vs* last CT	234, 66.3 ± 8.9	18.39	19.35	+0.97 (0.64–1.3)	<0.0001	1.24	−0.67	−1.88 (−2.99 to −0.77)	<0.0001
NFAI	132, 64.4 ± 9.3	16.54	17.26	+0.72 (0.31–1.13)	0.001	−0.56	−1.52	−0.96 (−2.46 to 0.54)	0.209
ACS	102, 67.2 ± 8.3	20.31	21.6	+1.29 (0.82–1.76)	<0.0001	3	−0.04	−3.04 (−4.73 to −1.35)	0.001
Baseline HU^m ≤^10	185, 66.7 ± 8.8	18.60	19.39	0.79 (0.43–1.14)	<0.0001	−3.05	−4	−0.94 (−2.07 to 0.19)	0.105
Baseline HU^m^ >10	49, 64.4 ± 9.7	17.59	19.2	1.61 (0.82–2.41)	<0.0001	17.40	11.97	−5.43 (−8.42 to −2.44)	0.001

NFAI, nonfunctioning adrenal incidentaloma; CT, computed tomography; ACS, autonomous cortisol secretion; Δ, difference; 95% CI, 95% confidence interval; HU^m^, mean of Hounsfield Unit in unenhanced CT.

Mean attenuation value at last visit was −0.7 HU^m^ (SD ± 10.7 HU^m^, range −28.4 to 30.1, Δ −2 HU^m^ *versus* baseline). An increase in attenuation value was observed in 73 adenomas (out of 234, 31%, range 0 to +19.8 HU^m^), 32 were patients with ACS. An increase ≥10 HU^m^ was observed in 16 adenomas (range +10 to +19.8 HU^m^, baseline attenuation value −27.8 to 20 HU^m^), five were ACS patients. Size and lipid content at the last visit were similar to baseline when CT was performed early (in 12–36 months), while differences increased over time, with the greatest change (Δ diameter +2 mm and Δ attenuation value −4 HU^m^) in case of long-term follow-up (>60 months in 101 cases, as described in **Table 2**). The reduction of lipid content was observed in NFAI and in ACS (respectively HU^m^ in the follow-up CT was lower than baseline in 52% and in 69% of patients, respectively, as reported in **Table 1**).

**Figure 2:**
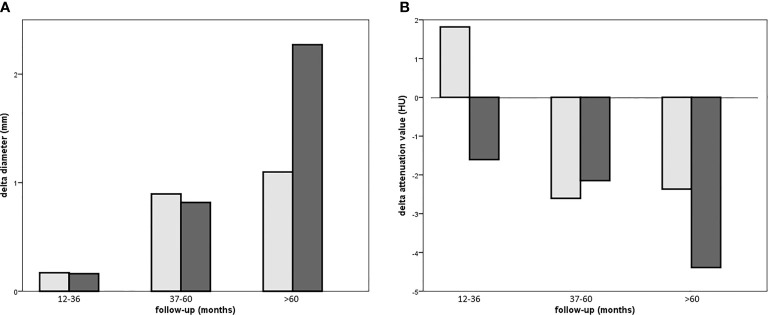
Bar chart depicting Δ diameter **(A)** or Δ attenuation **(B)** in patients with NFAI (light gray bar) or ACS (dark gray bar) according to short, intermediate, or long-term follow-up.

A positive linear regression was found between the duration of follow-up and Δ diameter (*r* = 0.289, *p* < 0.0001) and a negative regression between the duration of follow-up and Δ attenuation value (*r* = −0.195, *p* = 0.003, reassumed in **Figure 2**).

Considering endocrine secretion, during follow-up, an increase in diameter was observed either in patients with NFAI and in those with ACS (as reported in **Table 2**); however, a reduction in attenuation value was evident only in ACS, especially in the long-term follow-up (as depicted in **Figure 2**). Likewise, 69% of patients with ACS presented a lower attenuation value at follow-up CT with respect to baseline (*X* ^2^ = 6.371, *p* = 0.013, reported in **Table 1**).

Patients with lipid-poor adenoma (>10 HU^m^ at baseline CT scan) were more often female (postmenopausal, according to the age of onset), they presented a reduced cortisol suppression after 1-mg DST (see **Table 3**), an increase in size (as those with NFAI) and the largest decrease in attenuation value during follow-up (Δ −5.4 HU^m^).

**Table 3:** Clinical and radiological data of patients, sorted by attenuation value.

	HU^m ≤^10	HU^m^ >10	*p*-value
Age at diagnosis (years)	65.5 ± 2.9	62.9 ± 4.1	0.068
Gender female/male (% female)	119/105 (53%)	39/14 (74%)	0.001
Basal ACTH (ng/L)	17.15 ± 12.3	15.15 ± 9.59	0.236
Cortisol after 1-mg DST	59.6 ± 51.9	94.2 ± 80.1	<0.0001
UFC (nmol/24 h)	0.58 ± 0.22	0.59 ± 0.24	0.833
HbA1c (mmol/mol)	42.77 ± 9.23	41.7 ± 7.66	0.432
BMI (kg/m^2^)	29.61 ± 5.01	28.36 ± 5.36	0.197
Mean diameter (mm)	18.73 ± 6.98	18.71 ± 6.83	0.982
Δ diameter (mm)	0.8 ± 2.44	1.62 ± 2.77	0.044
Δ attenuation value (HU)	−0.94 ± 7.84	−5.43 ± 10.41	0.001
HU^m^ follow-up > baseline (%)	96 (43%)	17 (33%)	0.203
HU^m^ follow-up < baseline (%)	128 (57%)	36 (67%)	
Hypertension (%)	181 (81%)	39 (73%)	0.159
Diabetes mellitus (%)	45 (20%)	12 (22%)	0.645
Dyslipidemia (%)	101 (45%)	23 (43%)	0.767
Osteoporosis (%)	25 (11%)	8 (15%)	0.347

Data are presented as mean and standard deviation. NFAI, nonfunctioning adrenal incidentaloma; ACS, autonomous cortisol secretion; HU^m^, mean of Hounsfield unit in unenhanced CT; UFC, urinary free cortisol; BMI, body mass index; HbA1c, glycated hemoglobin; Δ, difference from baseline CT; DST, dexamethasone suppression test.

In order to predict the growth of an incidentaloma, we have divided the follow-up cohort according to their quartile of diameter: the groups obtained were homogeneous for number of adenomas, as reported in **Table 4**. ANOVA analysis (considering attenuation value, mean diameter, Δ difference, age, cortisol after 1-mg DST, baseline ACTH and UFC) revealed that cortisol after 1-mg DST at baseline (*F* = 11.09; *p* < 0.0001) or at last available visit (*F* = 11.639, *p* < 0.0001) and Δ diameter (*F* = 5.251, *p* = 0.002) were significant for the variation. Larger adenomas presented reduced suppression after 1-mg DST and greater increase in size during the follow-up, according to the *post-hoc* Dunnet’s test reported in **Table 5**.

**Table 4:** Radiological data of patients with a follow-up available CT, grouped by quartile of mean diameter (reported in the first column).

Quartile (diameter in mm)	*n*	Diameter baseline (mm)	Diameter follow-up (mm)	Δ Diameter (mm, 95% CI)	*p*-value *vs*. baseline	Attenuation value baseline (HU^m^)	Attenuation value follow-up (HU^m^)	Δ Attenuation value (HU^m^, 95% CI)	*p*-value *vs*. baseline
1st (8–14.1)	60	11.68	11.92	+0.25 (−0.26 to 0.52)	0.075	0.22	−0.59	−0.81 (−2.77–1.15)	0.413
2nd (14.1–18)	60	15.59	16.29	+0.7 (0.25–1.15)	0.003	0.61	−0.35	−0.96 (−3.42 to 1.49)	0.435
3rd (18.1–23.88)	56	19.85	20.8	+0.95 (0.18–1.72)	0.016	4.29	1.37	−2.92 (−5.36 to −0.49)	0.02
4th (23.9–42.2)	58	26.82	28.82	+1.98 (1.06–2.91)	<0.0001	1.01	−3.95	−2.94 (−5.09 to −0.78)	0.008

Δ, difference; 95% CI, 95% confidence interval; HU^m^, mean of Hounsfield Unit in unenhanced CT.

**Table 5:** Mean differences according to the quartile of diameter.

	Quartile	Mean difference *vs*. 1st quartile (95% CI)	*p*-value
Cortisol after 1-mg DST (nmol/L)	2nd (14.1–18 mm)	10.46 nmol/L (−14.84 to 35.77)	0.637
	3rd (18.1–23.88 mm)	19.23 nmol/L (−7.24 to 45.69)	0.207
	4th (23.9–42.2 mm)	60.08 nmol/L (34.03–6.1)	<0.0001
Diameter (mm)	2nd (14.1–18 mm)	0.46 mm (−0.6 to 1.53)	0.604
	3rd (18.1–23.88 mm)	0.72 mm (−0.363 to 1.81)	0.273
	4th (23.9–42.2 mm)	1.74 mm (0.67–2.82)	<0.0001

95% CI, 95% confidence interval.

Mean diameter in patients with adrenal CS was similar to those with ACS and larger than those with NFAI: 28.4 ± 6.63 mm, 24.48 ± 9 mm, and 18.98 ± 7.08 mm (respectively *p* = 0.231 and *p* = 0.005). Attenuation value was higher in adrenal CS than in ACS and NFAI: 22.28 ± 13.54 HU^m^, 2.16 ± 12.97 HU^m^, and −0.84 ± 11.34 HU^m^ (respectively *p* = 0.004 and *p* = 0.002), confirming the correlation between density of the adenoma and cortisol secretion. Univariate analysis confirmed these results (*F* = 21.162, *p* < 0.001 for diameter and *F* = 14.968, *p* < 0.001 for attenuation value), and *post-hoc* Dunnet’s test revealed that the difference in attenuation value was mild between ACS and NFAI (Δ difference 3 HU^m^, *p* = 0.027) and more evident between adrenal CS and NFAI groups (Δ difference 23.11 HU^m^, *p* < 0.0001).

## Discussion

Adrenal masses, discovered incidentally during an imaging study not performed for the suspicion of adrenal-related diseases, are increasingly detected in the adult-elderly population. Their management is not a minor concern for patients and healthcare-related costs. After comprehensive radiological (to exclude adrenal or metastatic malignancies) and endocrine evaluation (to assess excessive cortical or medullary secretion), a conservative management is proposed in patients with NFAI (1, 2, 23).

It has been extensively reported that there is a tendency to grow (9, 14, 15) or to develop ACS (6, 7, 9–12, 14, 15, 28, 29) in a proportion of patients with adrenal incidentaloma during follow-up. The ESE-ENS@T guidelines suggest no further evaluation if basal radiological assessment is consistent with a benign cortical adenoma (as diameter <4 cm and attenuation value <10 HU) (1). According to these guidelines, one study reported a minimal growth (1 mm) in 54 patients with benign incidentalomas after follow-up, suggesting that a radiological and endocrine reassessment can be performed after 5 years (30). In 2017, a large Korean study proposed that a diameter of 3.4 cm and attenuation value of 20 HU are able to distinguish benign adenoma from adrenal malignancy, without change in size in patients with NFAI (31). Attenuation value at CT is a feature that indicates lipid content in adrenal adenoma: it has been evaluated mostly at the baseline CT, and no data are available in longitudinal series. Therefore, the aim of our study was to collect radiological data (diameter and attenuation value) in a large cohort of patients with a long-term follow-up CT, according to cortisol secretion.

We describe a baseline cohort of 277 patients (with 355 different adenomas), and a follow-up group of 181 patients (234 adenomas) with available unenhanced CT after median 52 months (range 12–175 months, >60 months in 101 out of 181).

We confirmed that, if we consider radiological features consistent with a cortical adenoma [as diameter <10 cm and reduced attenuation value (32)], the overall growth of the adrenal mass was minimal, as recently reported (30, 31). We did not observe an increase in size if follow-up CT was performed early (<3 years after baseline imaging). Growth of the adenoma was significant (albeit modest) if the follow-up CT was performed after at least 5 years from diagnosis [close to 2 mm, as reported in a meta-analysis (17)]. Moreover, a positive linear regression was found between the duration of follow-up and increase in diameter: the longer the follow-up, the greater the growth, irrespective of endocrine secretion. Larger adenomas (>24 mm, the fourth quartile of diameter distribution) presented the greatest increase and the reduced cortisol suppression after 1-mg DST: the same threshold has been proposed by Morelli et al. for the risk of ACS development (12). As previously reported in several papers, we confirm a minimal growth and a tendency to develop ACS in case of long-term follow-up (6, 7, 9–12, 14, 15, 28, 29).

The change of the attenuation value during follow-up has been explored in our study. At baseline, mean density in patients with ACS was higher than in those with NFAI: reduced attenuation value indicate poor lipid content in patients with cortisol secretion (33). Overall, 66 incidentalomas presented at baseline an attenuation value >10 HU^m^ (reduced to 49 cases at the last CT evaluation), characterizing a lipid-poor adenoma: contrast-enhanced CT or MR were used to confirm the benign behavior of the mass, and a conservative management was indicated after multidisciplinary discussion (3). We confirmed a tendency to decrease the attenuation value, previously reported by Hammarstedt et al. in a smaller study (34), especially after a long-term follow-up: we observed a reduction of the attenuation value (−4 HU^m^) in 101 cases at least after 60 months. A negative regression between the duration of follow-up and attenuation value was observed, especially in patients with ACS: up to 70% of them presented a mean density lower than baseline. The largest decrease in attenuation value (−5 HU) was observed in those patients with mean density >10 HU^m^ at baseline CT scan. Intracellular lipid content can be studied with MR chemical shift: the quantitative assessment of adrenal-to-spleen ratio and signal intensity index are able to distinguish patients with ACS (35). Further studies, ideally prospective, are needed to clarify the association between lipid content and cortisol secretion in patients with adrenal adenoma. Age was not able to predict size or lipid content; however, according to our data, we can speculate a kind of “adrenal aging”. We described a growth in size and a reduction of the attenuation value during years of follow-up, irrespective of age at presentation, reflecting an increased lipid content of the adenoma. “Adrenal aging” is only a food-for-thought consideration and need to be further validated with histological analysis and prospective radiological studies.

We also collected imaging data from eight patients with overt monolateral adrenal masses secreting cortisol. Their diameter was similar to those with ACS, both larger than NFAI group, therefore adrenal size could not help clinicians in indicating cortisol secretion in patients with unsuppressed serum cortisol after 1-mg DST. On the contrary, attenuation value was higher in adrenal CS than in ACS, confirming the correlation between density (or lipid content) and cortisol secretion (33). As a matter of fact, HU^m^ was higher in CS, but significantly decreased in patients with ACS, and we can speculate that lipid content can be used to suggest an overt CS in patients with an adrenal adenoma and some peculiar features of hypercortisolism or cortisol-related comorbidities.

Our work presents some limitations. First, the observational longitudinal design of the study, without interventions: imaging follow-up in the absence of adrenalectomy is likely the general practice. Conservative management was one of the inclusion criteria: we included adrenal incidentaloma with a low-risk of progression based upon radiological features. According to our definition, also larger adenomas were excluded, because we selected those patients with benign adrenal incidentaloma, reflecting the suggestions of the ESE-ENS@T guidelines. Moreover, CT scans were performed as indicated during an outpatient visit, therefore follow-up was not homogenous. Our selection criteria reflect the clinical practice; however, a prospective and controlled study could help to further analyze the correlation between adrenal dimension, lipid content, and cortisol secretion.

To conclude, growth rate is significant but clinically modest in patients with adrenal incidentaloma selected with strict criteria indicating a benign behavior. According to our data, the first long-term imaging control with an unenhanced control CT can be proposed to patients with ACS or diameter >24 mm, at least 5 years after baseline. Lipid content, measured with attenuation value, is reduced in patients with ACS. In the follow-up, despite an increase in size, we observed a tendency to the reduction of mean density in adrenal incidentalomas.

## Data Availability Statement

The datasets presented in this study can be found in online repositories. The names of the repository/repositories and accession number(s) can be found below: http://researchdata.cab.unipd.it/id/eprint/530.

## Ethics Statement

The studies involving human participants were reviewed and approved by Ethics Committee of Padova University Hospital (Comitato Etico per la Sperimentazione Scientifica). The patients/participants provided their written informed consent to participate in this study.

## Author Contributions

FCe, IT, and FCr: writing—original draft, review, and editing. GV and GM: data acquisition and curation. IM, EQ, and CS: supervision and writing—review and editing. All authors contributed to the article and approved the submitted version.

## Funding

This study is supported by Grant “DiSCOG—University of Padova—Bando Pubblicazioni 2021” for APC.

## Conflict of Interest

The authors declare that the research was conducted in the absence of any commercial or financial relationships that could be construed as a potential conflict of interest.

## Publisher’s Note

All claims expressed in this article are solely those of the authors and do not necessarily represent those of their affiliated organizations, or those of the publisher, the editors and the reviewers. Any product that may be evaluated in this article, or claim that may be made by its manufacturer, is not guaranteed or endorsed by the publisher.
